# A Radiomics Model for Predicting Early Recurrence in Grade II Gliomas Based on Preoperative Multiparametric Magnetic Resonance Imaging

**DOI:** 10.3389/fonc.2021.684996

**Published:** 2021-09-02

**Authors:** Zhen-hua Wang, Xin-Lan Xiao, Zhao-Tao Zhang, Keng He, Feng Hu

**Affiliations:** Department of Radiology, The Second Affiliated Hospital of Nanchang University, Nanchang, China

**Keywords:** radiomics, grade II gliomas, MRI, multiparametric, recurrence

## Abstract

**Objective:**

This study aimed to develop a radiomics model to predict early recurrence (<1 year) in grade II glioma after the first resection.

**Methods:**

The pathological, clinical, and magnetic resonance imaging (MRI) data of patients diagnosed with grade II glioma who underwent surgery and had a recurrence between 2017 and 2020 in our hospital were retrospectively analyzed. After a rigorous selection, 64 patients were eligible and enrolled in the study. Twenty-two cases had a pathologically confirmed recurrent glioma. The cases were randomly assigned using a ratio of 7:3 to either the training set or validation set. T1-weighted image (T1WI), T2-weighted image (T2WI), and contrast-enhanced T1-weighted image (T1CE) were acquired. The minimum-redundancy-maximum-relevancy (mRMR) method alone or in combination with univariate logistic analysis were used to identify the most optimal predictive feature from the three image sequences. Multivariate logistic regression analysis was then used to develop a predictive model using the screened features. The performance of each model in both training and validation datasets was assessed using a receiver operating characteristic (ROC) curve, calibration curve, and decision curve analysis (DCA).

**Results:**

A total of 396 radiomics features were initially extracted from each image sequence. After running the mRMR and univariate logistic analysis, nine predictive features were identified and used to build the multiparametric radiomics model. The model had a higher AUC when compared with the univariate models in both training and validation data sets with an AUC of 0.966 (95% confidence interval: 0.949–0.99) and 0.930 (95% confidence interval: 0.905–0.973), respectively. The calibration curves indicated a good agreement between the predictable and the actual probability of developing recurrence. The DCA demonstrated that the predictive value of the model improved when combining the three MRI sequences.

**Conclusion:**

Our multiparametric radiomics model could be used as an efficient and accurate tool for predicting the recurrence of grade II glioma.

## Introduction

Glioma is a brain tumor originating from central glial cells with a high mortality rate (1–3). According to the World Health Organization (WHO), grade I and grade II tumors are classified as low-grade gliomas (LGG). LGGs are generally benign, with a recurrence rate of about 36% (4). Nevertheless, the clinical course of LGG may be unpredictable, as some of these tumors recur soon after primary treatment and/or undergo malignant transformation (5–7). A previous report indicated that low-grade gliomas (WHO II grade) have a 5-year survival rate of as high as 50% (8). Surgical resection followed by chemoradiation is the standard treatment option for gliomas. However, the risk and timing of recurrence following treatment in LGG are still difficult to predict accurately (9–12). Therefore, there is a need to identify accurate indicators for early detection and recurrence to provide timely, optimal treatment and improve survival.

Although histological analysis of surgical specimens is still considered the gold standard to grade gliomas, it may not always provide an accurate result (13) as the small sample obtained during the biopsy may not always reflect the grading heterogeneity within the entire tumor (14, 15). A substantial assessment would require the acquisition of samples from multiple regions within the tumor currently not widely accepted in clinical practice. Furthermore, a biopsy is an invasive procedure and also carries some risk. The acquisition of repeated biopsies is not always considered to be ethical as it may aggravate patient suffering.

The factors leading to poor OS post-surgery in LGG are still not well understood. Previous studies identified age, the extent of the tumor resection, and the expression of specific genes, including Ki-67 and the isocitrate dehydrogenase 1 (IDH1), as indicators for OS (16). Yet, to our knowledge, there is no accurate quantitative tool that could be used to predict at an early stage the risk of recurrence following the first tumor resection, highlighting the need to develop predictive models.

An alternative method that can be used to assess tumor recurrence post-surgery is magnetic resonance imaging (MRI). Previous studies have shown that radiomics could be used to quantitatively extract and assess numerous imaging features to effectively differentiate between high and low-grade gliomas (17, 18) and differentiate tumor recurrence from radiation necrosis (19). When combined with clinical data, imaging features could be used to assess the OS and hence optimize the treatment for the patient. Therefore, this study aimed to create a radiomics model based on clinical and imaging features to predict the risk of developing recurrence in grade II glioma after the first resection.

## Materials and Methods

### Participants

**Figure 1:**
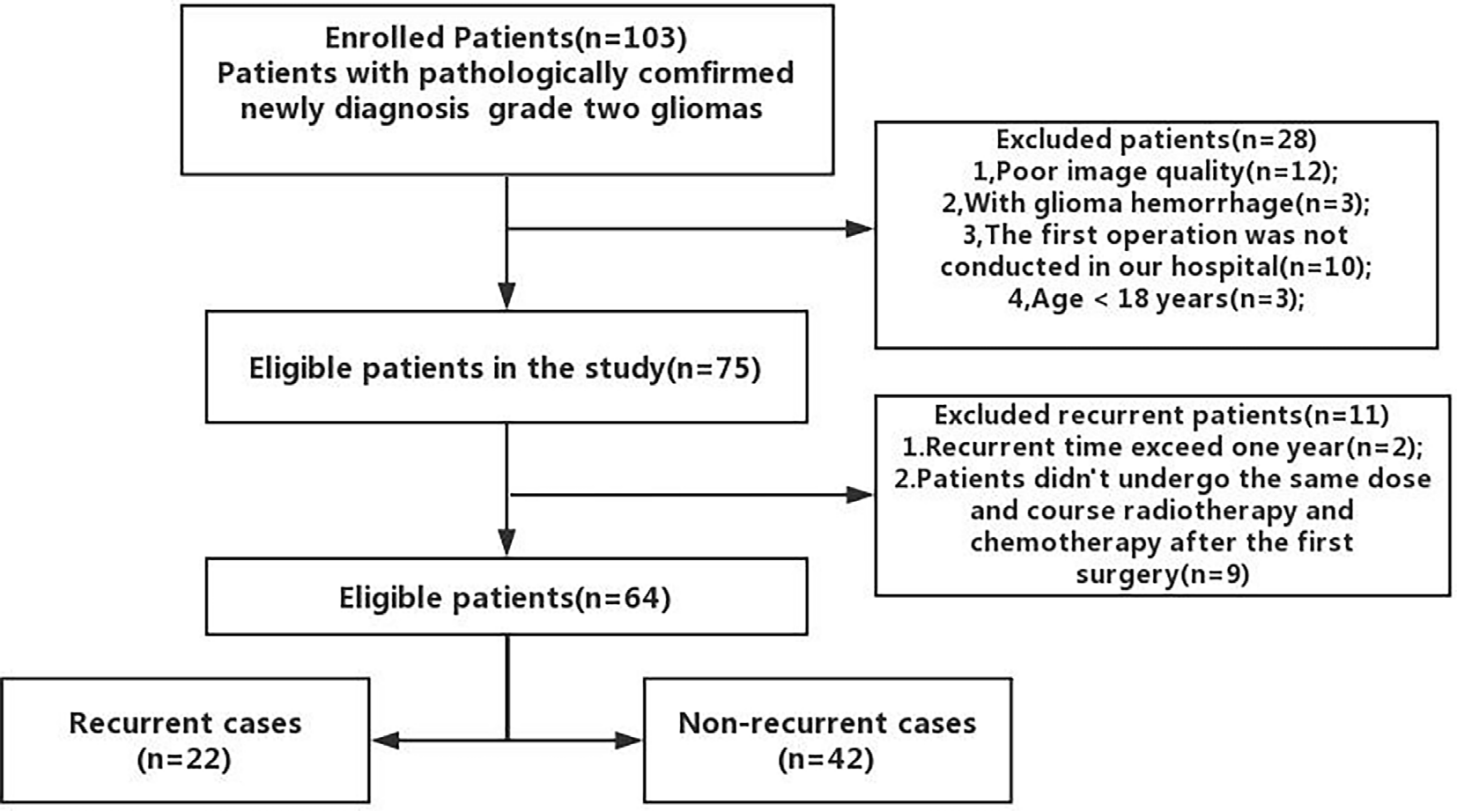
Flow diagram illustrating the patient selection process.

Retrospective analyses were performed on the follow-up medical records of 103 adult patients with histologically confirmed supratentorial grade II gliomas (according to WHO 2016 classification). All patients who had their first extensive glioma resection between May 2017 and November 2019 were included in the study. All patients had a MRI T1-contrast enhanced (T1CE) examination within 72 h after surgery to exclude the presence of a conspicuous residual tumor after surgery and received the same adjuvant chemoradiation treatment using a radiotherapy dose of 50.4 Gy in 28 fractions and 75 mg/m^2^ of temozolomide orally (20). Patients below 18 years with poor MRI images and tumor hemorrhage were excluded from the study (**Figure 1**). A total of 64 patients were ultimately included in the study.

### Data Collection

After being discharged, the patients were regularly followed up by the neurosurgery group of the hospital. A periodical MRI examination was performed after treatment, and any tumor progression was noted in the patient’s medical records according to the neuro-oncology (RANO) criteria (21). A biopsy was performed in those patients who had an obvious tumor progression noted on the MRI to further confirm the findings. The age, sex, progression-free survival (PFS), Ki-67, and IDH1 mutations were obtained from the patients’ medical records. Three magnetic resonance imaging (MRI) sequences, including T1-weighted (T1W1), T2-weighted (T2WI), and T1-contrast enhanced (T1CE), were acquired.

### MRI Parameters

All the patients underwent multi-sequence imaging protocol on a 3.0 Tesla MRI system (Discovery 750; GE Healthcare, Milwaukee, WI, USA), with an eight-channel head coil (GE Healthcare, Chicago, IL, USA). For the T1-weighted image (T1WI) acquisition, the repetition time/echo time (TR/TE), matrix size, field-of-view (FOV), slice thickness, slice gap, and acquisition time were 1,750/25.4 ms; 512 × 512, 220 × 220, 5 mm, 1.5 mm, and 89 s, respectively. For the T2WI acquisition, the (TR/TE), matrix size, FOV, layer thickness, layer spacing, and the number of layers were 4600/102 ms, 224 × 320, 220 × 220, 6 mm, 1, and 18, respectively. The axial T1CE sequence was acquired by repeating the T1WI described above after a bolus injection of 0.1 mmol/kg of gadodiamide (Omniscan, GE Healthcare, Cork, Ireland).

### Description of the Region of Interest and Assessment of the MRI Sequences

The ITK-snap software (www.itk-snap.org) was used to analyze the MRIs. A region of interest (ROI) was blindly delineated by two senior radiologists with more than 10 years of work experience. The boundaries of most low-grade tumors without contrast enhancement were determined on the T2WI images as these images are widely accepted in the identification of hyperintense signals representing the tumor regions (22). Then, the contours of the tumor delineated on the T2WI were transferred to the T1WI and T1CE images. In tumors with contrast enhancement, the tumor boundaries were delineated on the T1CE images by selecting the enhanced region. The delineated region was transferred onto the T1WI and T2WI images.

**Figure 2:**
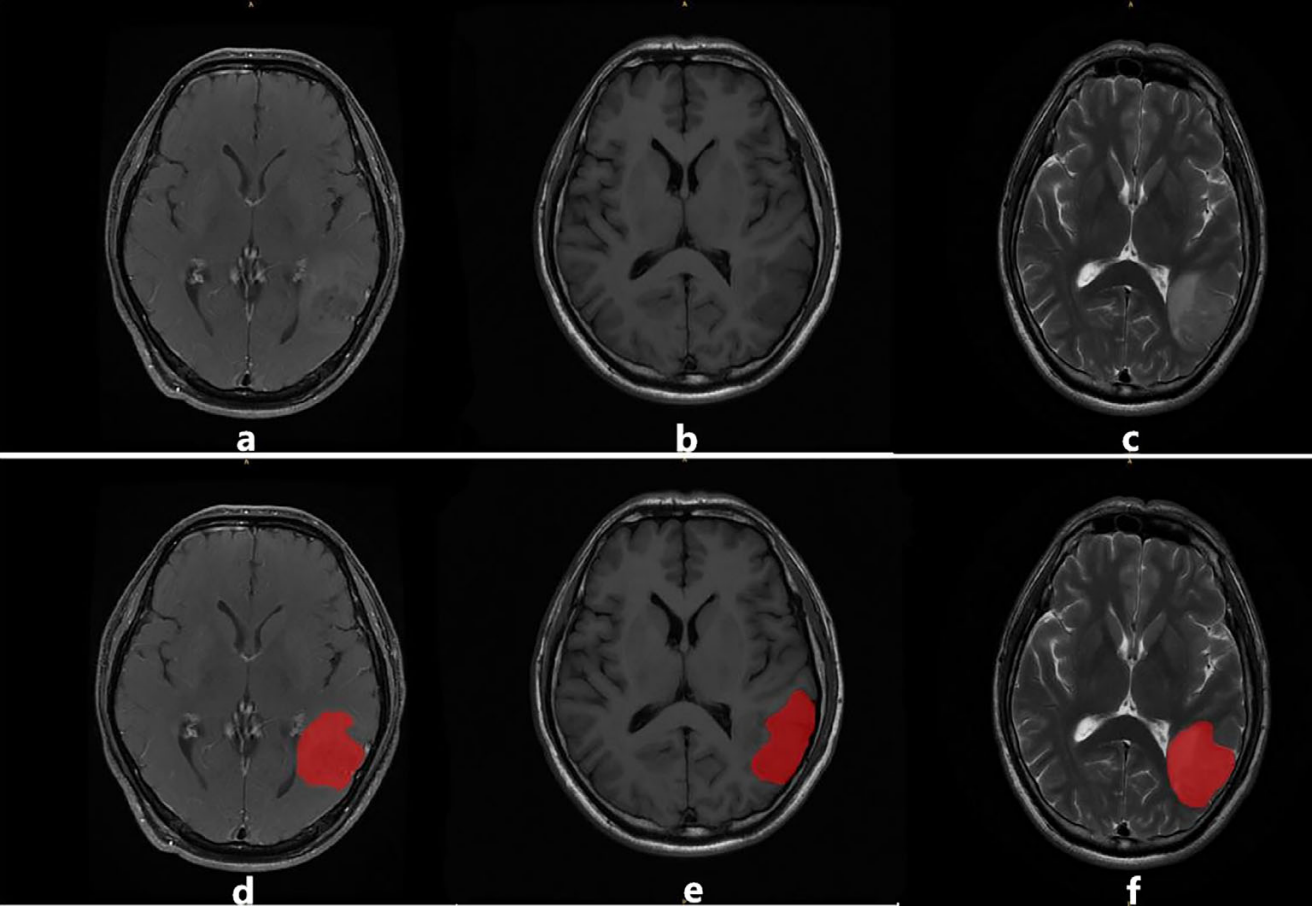
An example of image segmentation: **(A–C)** illustrate T1CE, T1WI, and T2WI sequences, respectively. Images **(D–F)** illustrate the region of interest (ROI) in red delineated by the radiologists for feature extraction.

After the delineation of the ROI, all the patients were divided into the recurrent group (RG) and non-recurrent group (NRG) based on the RANO criteria (indicated in **Table 1**) and biopsy findings by two radiologists. In case of any disagreement, a consensus was reached through discussion, especially when there was a discrepancy between the two readers, as illustrated in **Figure 2**.

**Table 1:** RANO criteria used to evaluate treatment response in low-grade gliomas.

Criterion	Complete remission	Partial remission	Stable disease	Progress disease
T1CE	Not seen	Decrease ≥50%	Increase or decrease in the range of -25% ~ +25%	Increase ≥ 25%*
T2WI/FLAIR	Stable or diminished	Stable or diminished	Stable or diminished	Increase*
New lesion	None (apart from those consistent with radiation effects, and no new or increased enhancement)	None (apart from those consistent with radiation effects, and no new or increased enhancement)	None (apart from those consistent with radiation effects, and no new or increased enhancement)	Present*
Corticosteroids	None	Stable or diminished	Stable or diminished	Not apply
Clinical status	Stable or improved	Stable or improved	Stable or improved	Deteriorative*(not attributable to other causes apart from the tumor, or decrease in corticosteroid dose)
Requirement for response	All	All	All	Any

CE, contrast-enhanced; FLAIR, fluid-attenuated inversion recovery.

*Progress is determined by anyone project.

### Feature Extraction

Radiomic features were extracted using the AK software (Artificial Intelligence Kit V3.0.0.R, GE Healthcare). A total of 396 features were extracted from each MRI sequence, including the Laplacian of Gaussian (LoG), rotation invariant local binary patterns (RILBP), the gray level co-occurrence matrix (GLCM), intensity-based features (IBF), directional Gabor texture features (DGTF), and rotation invariant circular Gabor features (RICGF), etc. These features were then used to construct the multiparametric model.

### Data Preprocessing and Feature Screening

The dataset was randomly categorized into the training or validation set using a ratio of 7:3. All cases in the training set were used to train the predictive model, while cases in the validation set were used to evaluate the model’s performance independently. Variables with zero variance were excluded from the analysis. The missing values were substituted with the median value. Finally, the z-score was used to standardize the data (23). Feature screening was performed by using the minimum redundancy-maximum relevance (mRMR) (24) method alone or in combination with univariate logistic analysis. A *p*-value below 0.05 was deemed statistically significant.

### Development and Validation of Models

Logistic regression analysis was used to construct predictive models based on the extracted optimal feature subsets of the training dataset. A receiver operator curve (ROC) was used to assess the performance of the radiomics models, and the sensitivity, specificity, and area under the curve (AUC) were calculated using five-fold cross validation. Calibration curves and decision curve analyses (DCA) were used to assess the clinical predictive performance of the models. The models were constructed using the R software (version 4.0.2), and a two-tailed *p*-value below 0.05 was deemed statistically significant.

### Statistical Analysis

According to the normality of samples based on the Shapiro-Wilk test, the independent samples t-test, the chi-square (x^2^) test, Fisher’s exact test and the Mann-Whitney U-test were used to identify any differences in age, gender, and other baseline characteristics between the training set and validation set. This data was analyzed using the statistical package for the social sciences (SPSS) version 22.0 software.

### Ethical Considerations

Ethical approval was obtained from our hospital ethics committee. The need to obtain informed consent from patients was waived due to the retrospective nature of the study.

## Results

### Patient Characteristics

The characteristics of the tumors and patients are summarized in **Table 2**. A total of 64 patients were included in the analysis. Following the first surgical resection, 64 patients were confirmed as grade II gliomas. According to the RANO criteria, 29 patients were thought to have a tumor recurrence and underwent a biopsy. The biopsy confirmed the recurrence in 22 patients, while the other 7 patients were diagnosed with pseudo-response.

**Table 2:** Baseline demographics and clinical characteristics of patients in the training and validation datasets.

Clinicopathological Variable		Training set (n=44)			Validation set (n=20)		
		NRG	RG	*p*-value	NRG	RG	*p-*value
Numbers of cases		30	14		12	8	
Age		40.60 ± 12.20	48.36 ± 9.74	0.047	39.77 ± 14.31	51.25 ± 8.12	0.053
Gender, n(%)	Female	15 (50)	6 (42.9)	0.659	4 (30)	4 (50)	0.648
	Male	15 (50)	8 (57.1)		8 (70)	4 (50)	
IDH1-mutation, n(%)	Wild-type	6 (20)	5 (35.7)	0.287	5 (41.7)	3 (37.5)	1.00
	Mutation-type	24 (80)	9 (64.3)		7 (58.3)	5 (62.5)	
Tumor crossing the midline, n(%)	Non	25 (83.3)	10 (71.4)	0.610	9 (75)	6 (75)	1.00
	Yes	5 (16.7)	4 (28.6)		3 (25)	2 (25)	
Ki-67 [median (IQR)]		5.0 (2.0-8.0)	5.5 (3.0-10.0)	0.533	5.0 (2.25-8.0)	7.0 (5.0-14.75)	0.238

NRG, Non-recurrent group; RG, recurrent group; IQR, interquartile range.

### Clinicopathological Characteristics

Among the 64 patients included in the study, 22 had a pathologically confirmed recurrent tumor, and the rest did not have any recurrence. The patients were randomly divided into training and validation datasets using a ratio of 7:3. The baseline characteristics of the subjects are summarized in **Table 2**. There was no significant difference in the age (*p* = 0.251), gender (*p* = 0.475), frequency of glioma recurrence (*p* = 0.845), Ki-67 (*p* = 0.486), and IDH1 (*p* = 0.885) mutation status and tumors crossing the midline (*p* = 0.307) between the training and validation group. There was a statistically significant difference (*p* < 0.05) in age between the RG and NRG in the training set. All other clinicopathological features did not differ significantly between the two groups.

### Performance of the Radiomics Models

**Figure 3:**
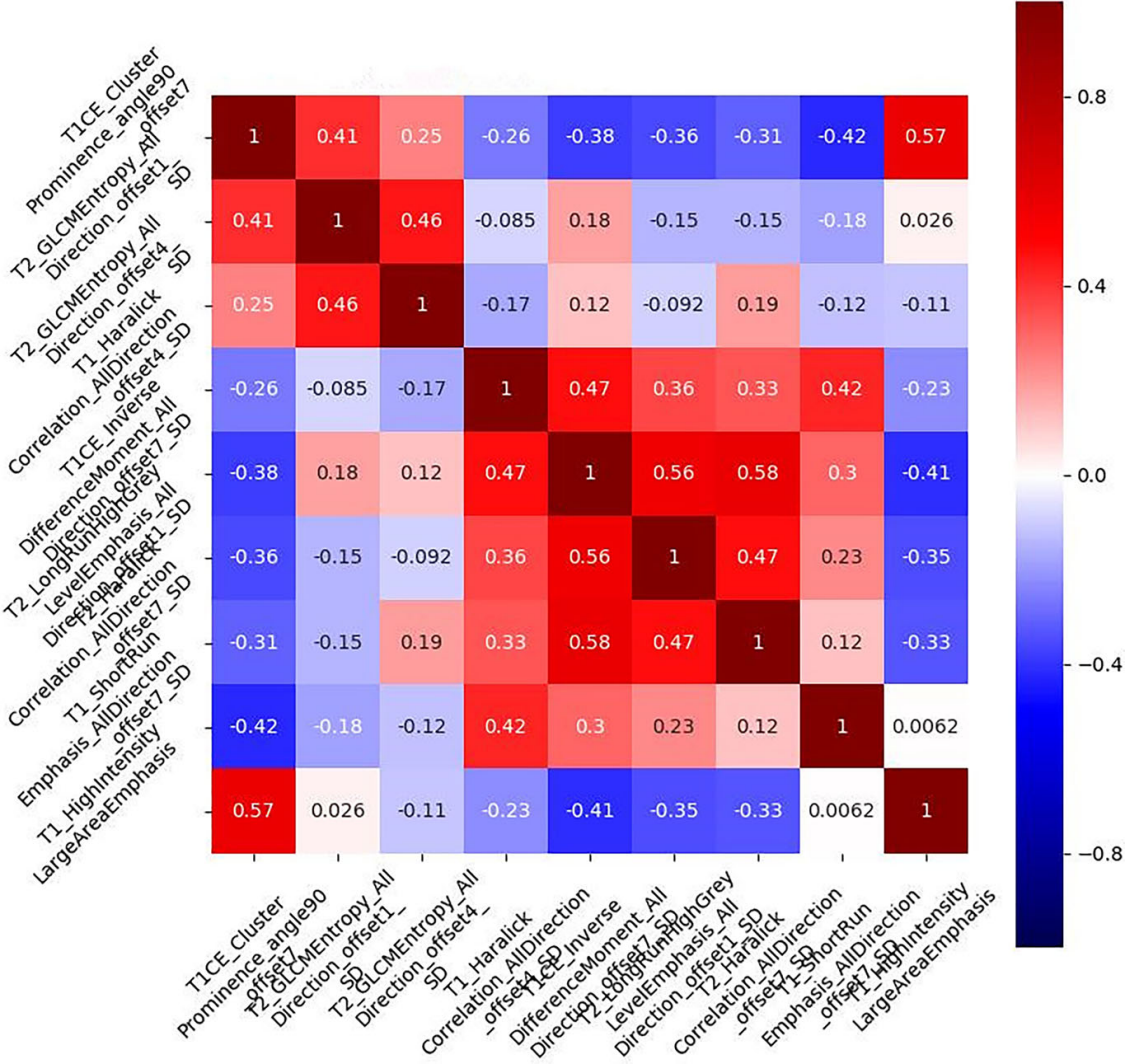
Correlation coefficient of the combined multiparametric models. The correlation coefficients of the selected nine features were low, indicating that the nine feature clusters were not redundant. The magnitude of the correlation is illustrated in the color bar on the right.

We extracted 396 features from the ROIs of every sequence. After running the mRMR algorithm, six features were selected from the T1WI images, five features from the T2WI images, and four features from the T1CE images. These three sequences were subsequently combined to identify the most important predictive features of the multiparametric model. Based on the univariate logistic analysis and mRMR, nine predictive features were eventually identified, and their correlation coefficients are illustrated in **Figure 3**. The low correlation coefficient between the nine features indicates little redundancy among every feature cluster.

**Figure 4:**
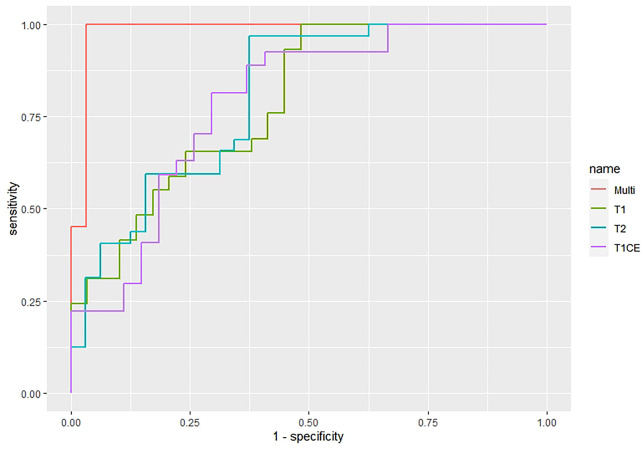
The ROC curves of the four imaging prediction models whereby the green curve represents the T1WI model, the blue curve represents the T2WI, the purple curve represents T1CE, and the red curve represents the multiparametric MRI model.

**Figure 5:**
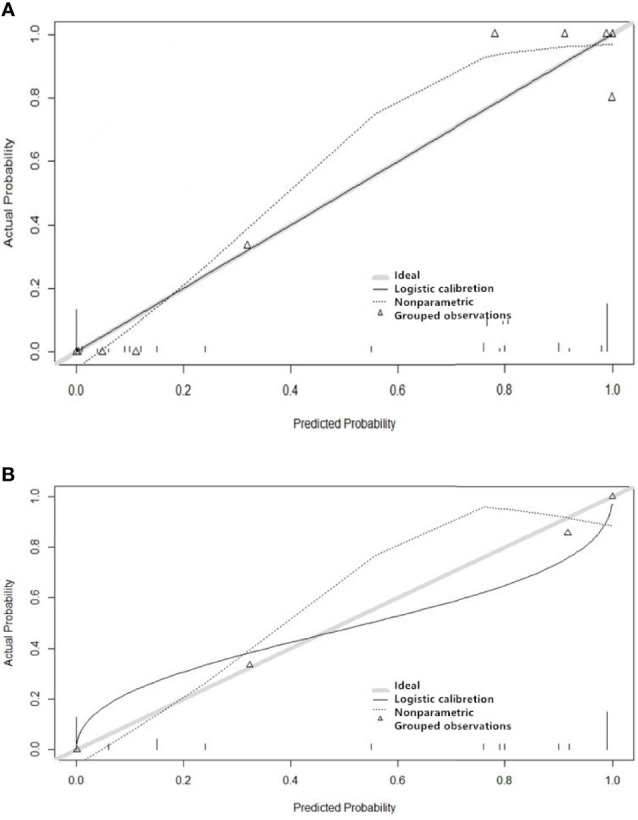
Performance of the four imaging models for predicting the recurrence of grade II gliomas. The y-axis represents the actual probability, and the x-axis represents the predicted probability. Figure **(A)** shows the model’s calibration of the training set, and Figure **(B)** shows the validation set. A calibration curve describes the consistency between the predicted and actual tumor recurrence rate. The 45° gray heavy lines represent the ideal prediction performance, the non-45dotted lines represent the prediction performance of the model, and non-45° solid lines represent the corrected prediction performance of the model. The closer the solid line is to the ideal gray line, the better the prediction accuracy of the model.

The features screened from the T1WI, T2WI, T1CE, and multiparametric sequences are summarized in **Table 3**. Four radiomics models were established for predicting tumor recurrence based on the screened optimal predictive features and their contributing predictive weight for each image sequence, as illustrated in **Table 3**. In the T1WI sequence, six predictive features were included in the model, eventually resulting in an AUC of 0.842 and 0.79 in the training and validation datasets, respectively. In the T2WI sequence, five predictive features were used to construct the models, resulting in an AUC of 0.785 in the training set and 0.790 in the validation set. In the T1CE sequence, four predictive features were used to develop the predictive model, which resulted in an AUC of 0.784 in the training set and 0.803 in the validation set. The multiparametric MRI model included nine predictive features from the T1WI, T2WI, and T1CE sequence, resulting in the best overall performance with an AUC of 0.966 and 0.930 for the training and validation datasets, respectively (**Table 4** and **Figure 4**). The calibration curves of the model also indicated a good agreement between the predicted probability and actual tumor recurrence both in the training set and validation set, indicating that the model was well-calibrated (**Figure 5**).

**Table 3:** The screened features and their coefficients in the models for the different imaging sequences.

Modality	Variables	Coefficient.	Sth.Err.	Z	*P* > |z|	[0.025	0.975]
T1CE	Intercept	-0.3825	0.5451	-0.7017	0.4828	-1.4508	0.6858
	GLCMEntropy_AllDirection_offset4_SD	0.5540	0.5905	0.9382	0.3481	-0.6034	1.7114
	Compactness2	0.6443	0.6101	1.0560	0.2910	-0.5515	1.8401
	ShortRunEmphasis_AllDirection_offset7_SD	-1.1118	0.9869	-1.1265	0.2600	-3.0461	0.8226
	LongRunEmphasis_angle0_offset1	-1.2584	0.7682	-1.6382	0.1014	-2.7640	0.2472
T1WI	intercept	0.0196	0.3750	0.0523	0.9583	-0.7154	0.7546
	GLCMEntropy_AllDirection_offset7_SD	0.9189	1.1705	0.7850	0.4324	-1.3753	3.2132
	LowGreyLevelRunEmphasis_AllDirection_offset1_SD	-3.9480	3.5082	-1.1254	0.2604	-10.8240	2.9279
	RunLengthNonuniformity_AllDirection_offset4_SD	0.3930	0.4920	0.7987	0.4245	-0.5714	1.3573
	ShortRunEmphasis_AllDirection_offset7_SD	-0.3207	0.4130	-0.7765	0.4375	-1.1302	0.4888
	ShortRunEmphasis_angle90_offset4	1.0657	2.3549	0.4525	0.6509	-3.5498	5.6812
	Variance	1.7391	0.7825	2.2225	0.0262	0.2054	3.2728
T2WI	intercept	-0.1973	0.4298	-0.4591	0.6461	-1.0397	0.6451
	ClusterShade_angle45_offset7	0.0706	0.3401	0.2075	0.8356	-0.5960	0.7372
	Correlation_AllDirection_offset1_SD	-0.4181	0.4751	-0.8800	0.3788	-1.3494	0.5131
	Sphericity	-0.6831	0.4032	-1.6941	0.0902	-1.4734	0.1072
	HighIntensityLargeAreaEmphasis	-0.0726	0.4370	-0.1662	0.8680	-0.9291	0.7838
	LongRunEmphasis_angle90_offset1	-1.4411	0.9134	-1.5777	0.1146	-3.2313	0.3491
Multiparametric	intercept	-0.1338	2.3732	-0.0564	0.9550	-4.7852	4.5176
	T1CE_ClusterProminence_angle90_offset7	-2.4287	2.7045	-0.8980	0.3692	-7.7295	2.8721
	T1CE_InverseDifferenceMoment_AllDirection_offset7_SD	2.3638	3.4494	0.6853	0.4932	-4.3970	9.1245
	T2_GLCMEntropy_AllDirection_offset1_SD	2.0994	2.7421	0.7656	0.4439	-3.2750	7.4739
	T2_GLCMEntropy_AllDirection_offset4_SD	-1.2254	0.0745	1.683	0.0924	-0.8635	1.5517
	T2_LongRunHighGrayLevelEmphasis_AllDirection_offset1_SD	0.9696	1.2682	0.7646	0.4445	-1.5161	3.4553
	T2_HaralickCorrelation_AllDirection_offset7_SD	-10.1476	6.6254	-1.5316	0.1256	-23.1332	2.8380
	T1_HaralickCorrelation_AllDirection_offset4_SD	-1.8642	1.6618	-1.1218	0.2620	-5.1213	1.3929
	T1_ShortRunEmphasis_AllDirection_offset7_SD	-1.9502	1.4283	-1.3654	0.1721	-4.7497	0.8493
	T1_HighIntensityLargeAreaEmphasis	2.6325	1.6886	1.5590	0.1190	-0.6770	5.9420

**Table 4:** The performance of the models for predicting tumor recurrence in the training and validation datasets.

Modality	Features screening method	Number of features after screened	Cohort	AUC (95% CI)	Sensitivity	Specificity	Accuracy
T1WI	mRMR	6	Training	0.842 (0.674–0.905)	0.7	0.7	0.75
			Validation	0.79 (0.687–0.902)	0.778	0.778	0.78
T2WI	mRMR	5	Training	0.785 (0.697–0.912)	0.727	0.682	0.705
			Validation	0.79 (0.679–0.92)	0.8	0.5	0.65
T1CE	mRMR	4	Training	0.784 (0.665–0.913)	0.889	0.556	0.722
			Validation	0.802 (0.693–0.911)	0.78	0.778	0.8
Multi-modalities	ULA + mRMR	9	Training	0.966 (0.949–0.99)	0.905	0.952	0.929
			Validation	0.93 (0.905–0.973)	1	0.8	0.90

ULA, univariate logistic analysis.

**Figure 6:**
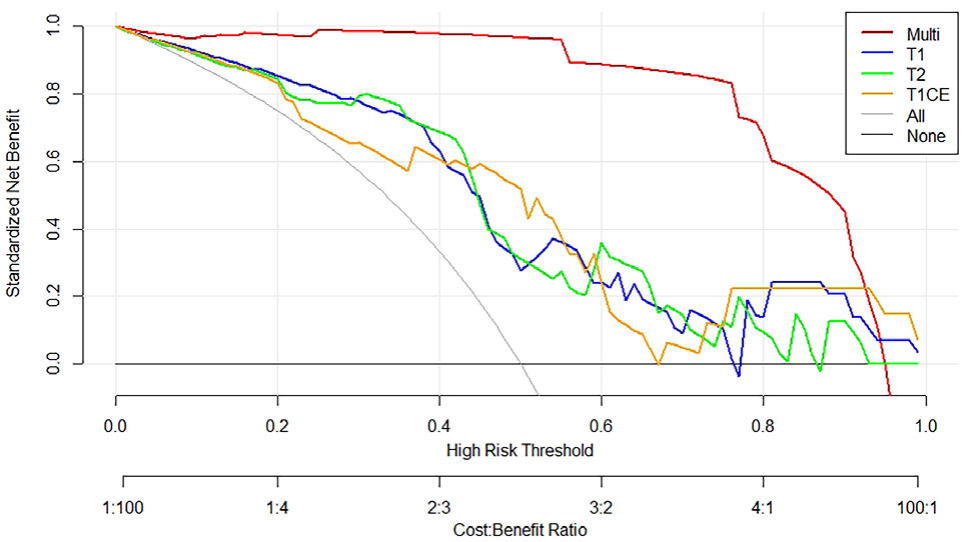
Decision curve analysis constructed using the radiomics features extracted from T1WI, T2WI, and T1CE sequences. The y-axis measures the net benefit of the T1WI (blue curve), T2WI (green curve), T1CE (yellow curve), and the multiparametric (red curve) images. The gray curve represents the assumption that all patients were treated, and the straight black line at the bottom of the figure represents the assumption that none of the patients were treated.

The DCA for the individual T1WI, T2WI, T1CE, and these combined multiparametric models are illustrated in **Figure 6**. The net benefit of the model constructed based on the three sequences was higher than the one based on the individual imaging sequence, to which it was superior across nearly the entire range of clinically useful threshold risks.

## Discussion

Surgery followed by chemoradiation is the main treatment option for patients diagnosed with LGG. Tumor recurrence post-treatment is one of the factors leading to poor OS. Surgical resection is one of the treatment options for patients diagnosed with recurrent LGG. Still, guidelines issued by several professional bodies state that there is limited high-level clinical evidence on the effectiveness of a secondary invasive resection on survival. A study by Patrizz et al. (25) indicated that histopathologic findings following chemoradiation do not always correlate with clinical outcomes in patients diagnosed with recurrence post-surgery. First of all, the pathological specimens may not always reflect the nature of the whole tumor. Furthermore, several studies found that other clinical factors that may have an impact on survival including age, radiotherapy dose, and the extent of tumor resection (16). Multiparametric MRI has played an important role in distinguishing between LGG and HGG as well as recurrence from radiation-induced necrosis. However, to our knowledge, currently, there is no suitable clinical and image-based predictive model to assess the risk of recurrence post-surgery in LGG patients. Therefore in this study, we made use of the imaging data of 64 LGG patients to develop a model that could be used to predict recurrence in these patients and hence enable clinicians to identify the patients that are most likely to benefit from additional surgery.

In our study, there was no difference in the baseline characteristics between the RG and NRG except for age. Consistent with the retrospective study by Li et al. (16), age was found to be an important risk factor for recurrence in grade II gliomas following the first surgery. Jansen et al. (26) conducted a long-term follow-up of 110 patients with LGG (WHO Grade II) after resection. Their results demonstrated that the initial extent of the resection influenced the progression-free survival, time to malignant transformation, and overall survival. Moreover, Patrizz et al. (25) indicated that the radiotherapy dose after surgery has a significant impact on survival in LGG patients. In our study, all patients had an extensive tumor resection and received the same radiation dose. Therefore, the effects of these variables on tumor recurrence could not be assessed.

Studies have shown a high correlation between certain genetic alterations, recurrence, and prognosis in grade II and III gliomas. Mutations of the isocitrate dehydrogenase (IDH)1/2 genes are common events in gliomas (27), especially among grade II gliomas, where IDH1 mutations are observed in about 70% to 80% of cases (27, 28). Some studies indicated that IDH1 mutation status could improve OS and PFS in grade II and III glioma (19, 29). Although the IDH1 mutation has been identified as an independent positive prognostic biomarker for survival in patients with glioma (26, 30), the association between the IDH mutant status and the risk of developing recurrence is still not clear. In the present study, the proportion of IDH mutation cases was noticeably higher in NRG compared with RG [31/42(73.8%) *vs* 14/22(63.6%)]; however, the statistic results showed that there was not a significant difference between NRG and RG (**Table 2**), which indicated that there might not be a link between the IDHI mutation and tumor recurrence; nevertheless, due to the limitation of our relatively small sample size, it still needs a big sample for further verification.

The RANO criteria are still widely used to assess the tumor response post-treatment and the need for additional treatment (31, 32). Despite being used extensively, the accuracy rate of the RANO criteria in distinguishing between tumor recurrence and pseudo-response (32, 33) in our study was only 75.86%. The multi-parameters radiomics model developed in our study resulted in higher prediction accuracy in both testing and validation datasets.

In order to develop our radiomics model, numerous features were extracted from each of the three MRI sequences. It is important to acknowledge that the sample size in our study was relatively small, potentially over-fitting the model (34). In order to reduce this risk, mRMR was used for feature dimensionality reduction. This technique has been widely used in several studies and involves selecting features from the mutually correlated distance or similarity score hence facilitating the data screening process (35, 36).

Numerous studies evaluated the use of radiomics models in predicting recurrence in glioma after radiotherapy. Wang et al. (37) proposed a radiomics model based on MRI and PET images to discriminate between tumor recurrence from radiation necrosis. The model performed well in both training and validation datasets with an AUC of 0.988 and 0.914, respectively. A similar model based on 51 glioma patients developed by Quan Zhang et al. (19) achieved outstanding performance with an AUC of 0.962 following validation. However, to the best of our knowledge, this is the first multiparametric model developed to predict recurrence in LGG before surgery. Our model also achieved an excellent performance, with an AUC of 0.966 and 0.930 in the testing and validation dataset, respectively.

In the study, a total of nine optimal features were selected for the construction of the multiparametric radiomics model. Among these features, there were three gray level run length matrix (GLRLM) features (T2_LongRunHighGrayLevelEmphasis_AllDirection_offset1_SD, T1_ShortRunEmphasis_AllDirection_offset7_SD, and T1_ShortRunEmphasis_AllDirection_offset7_SD), one gray level size zone matrix (GLSZM) feature (T1_HighIntensityLargeAreaEmphasis), and the rest were gray level co-occurrence matrix (GLCM) features (**Table 3**). The above results indicate that GLCM features played the most important role in the model. In some previous radiomics studies, the GLCM features also played an important role in predicting the IDH mutation status. Checkout et al. developed a new approach to predict IDH mutation status that outperformed competing methods (38), while Park et al. (39) found that GLCM was one of the strongest IDH status prediction factors. Furthermore, in a study by Chaddad et al. (40), GLCM had a significant role in predicting survival in patients with glioblastoma. Combined with these previous studies, we can reasonably infer that GLCM may convey information that could potentially be used to predict recurrence.

Both calibration and discrimination are valuable aspects of a prediction model (41). AUC is a common evaluation index of discrimination, while calibration reflects the level of agreement between the actual observed outcomes and the model’s predicted outcomes (42). However, the AUC focuses merely on the predictive accuracy of the signature. As such, it does not tell us whether the model is worth using at all. DCA is a statistical method that incorporate consequences and, thus, can inform the decision of whether to use this model (43). Therefore to further complement the AUC findings, a DCA was also performed to evaluate the clinical value of the models (44). In our study, both the AUC and calibration curve (**Figure 5**) showed that our model has a high prediction accuracy. Furthermore, the DCA curves showed that within a relatively large threshold range, our proposed radiomics models could be used to improve the treatment decision-making process. However, the DCA showed that multiparametric MRI models had a significantly higher performance when compared with models based on a single MRI sequence across nearly the entire range of clinically useful threshold risks (**Figure 6**).

This study has some limitations that have to be acknowledged. The majority of the patients with recurrent LGG at our institution generally prefer to be treated with radiotherapy and chemotherapy as opposed to surgery. This limited the sample size in our study and hence limited the number of clinical, pathological, molecular, and imaging features that could be used to train the model. In order to improve the robustness and generalizability of the model, further studies with a larger sample from multiple institutions with a longer follow-up are warranted. A larger sample will also allow us to apply different machine learning strategies to improve the prediction performance of the model. Further research is also recommended to illustrate the relationship between specific imaging features and pathology. Finally, additional studies are also recommended to evaluate the impact of early recurrence prediction on the provision of timely interventions and ultimately survival.

## Conclusion

The application of our radiomics model-based features extracted from multiparametric MRI could be used to predict the risk of early recurrence of grade II gliomas after the first surgical resection. This model could be used to guide the clinicians’ decision on the need for further invasive treatment such as biopsy and surgery in LGG patients.

## Data Availability Statement

The raw data supporting the conclusions of this article will be made available by the authors, without undue reservation.

## Ethics Statement

The studies involving human participants were reviewed and approved by The Second Affiliated Hospital of Nanchang University Medical Research Ethical Committee. Written informed consent for participation was not required for this study in accordance with the national legislation and the institutional requirements.

## Author Contributions

Conceptualization: Z-hW, X-LX, and KH. Data curation: Z-hW. Formal analysis: Z-hW, X-LX, and KH. Funding acquisition: Z-hW and X-LX. Investigation: Z-hW, X-LX, and FH. Methodology: Z-hW, X-LX, and Z-TZ. Project administration: Z-hW, X-LX, and KH. Resources: Z-hW, X-LX, and KH. Software: Z-hW, X-LX, and FH. Supervision: Z-hW, X-LX, and KH. Validation: Z-hW, X-LX, and Z-TZ. Visualization: Z-hW, X-LX, and Z-TZ. Writing—original draft: Z-hW and X-LX. Writing—review and editing: Z-hW and X-LX.

## Funding

This work was supported and funded by the Key research and development projects in Jiangxi Province, China (NO.20171ACG70002).

## Conflict of Interest

The authors declare that the research was conducted in the absence of any commercial or financial relationships that could be construed as a potential conflict of interest.

## Publisher’s Note

All claims expressed in this article are solely those of the authors and do not necessarily represent those of their affiliated organizations, or those of the publisher, the editors and the reviewers. Any product that may be evaluated in this article, or claim that may be made by its manufacturer, is not guaranteed or endorsed by the publisher.
